# Supplementary material for: CUC/auxin patterning of decanalised petal number in Cardamine hirsuta
Source: Quant Plant Biol. 2025 Jul 1;6:e29. doi: 10.1017/qpb.2025.10015 (PMC12451245; doi:10.1017/qpb.2025.10015)
Supplement: Rambaud-Lavigne et al. supplementary material [file S2632882825100155sup001.docx]

**Supplementary data**

**
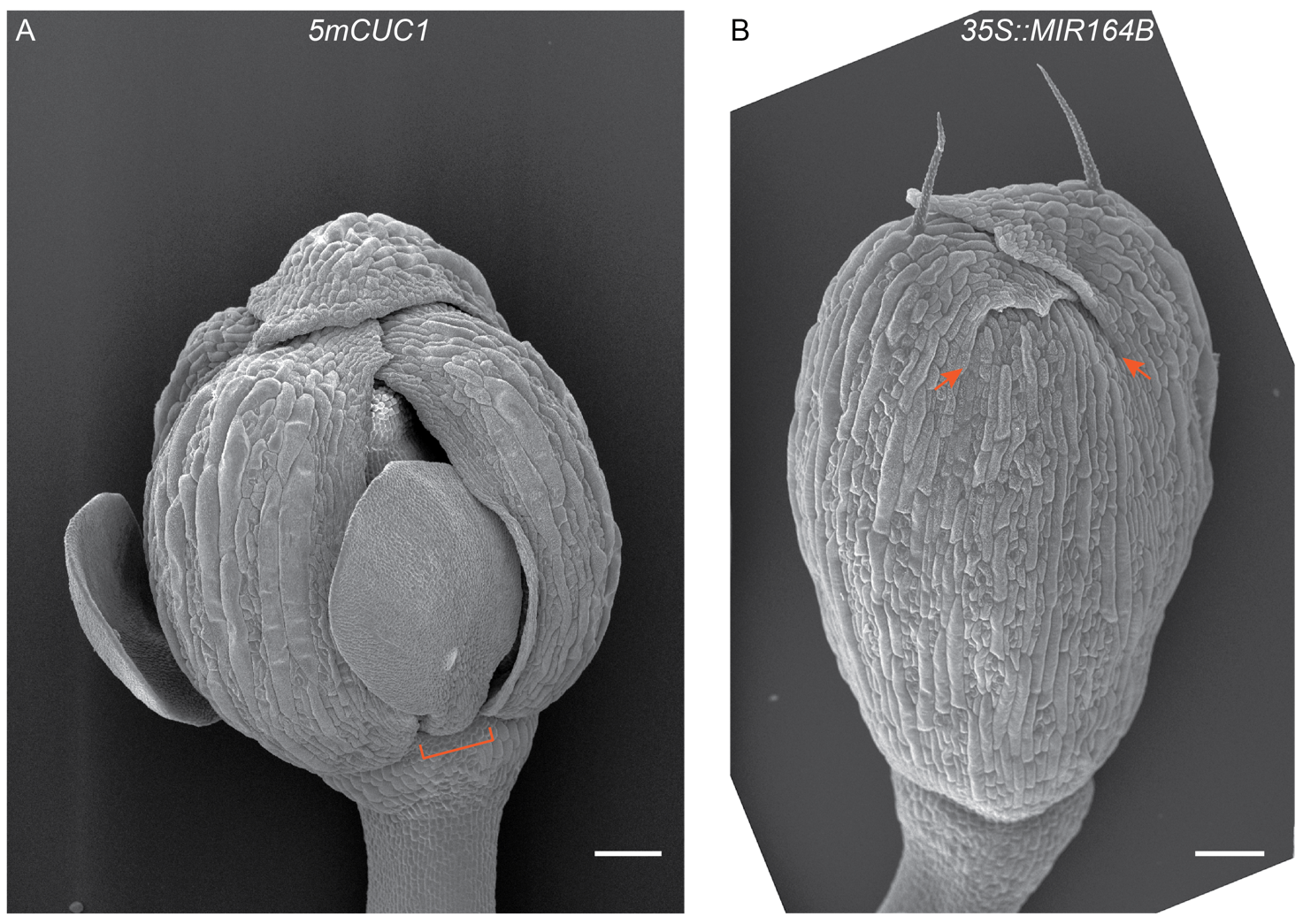
**

#### Fig. S1 Boundaries between sepals are affected in *C. hirsuta* *CUC* gain- and loss-of-function lines. Scanning electron micrographs of representative *C. hirsuta* *5mCUC1* (A) and *35S::MIR164B* (B) closed flower buds. Orange bracket in (A) indicates an enlarged boundary between sepals where a developing petal is visible. Orange arrows in (B) point to fusions between adjacent sepals, indicating loss of inter-sepal boundaries. Scale bars: 100 µm.

**
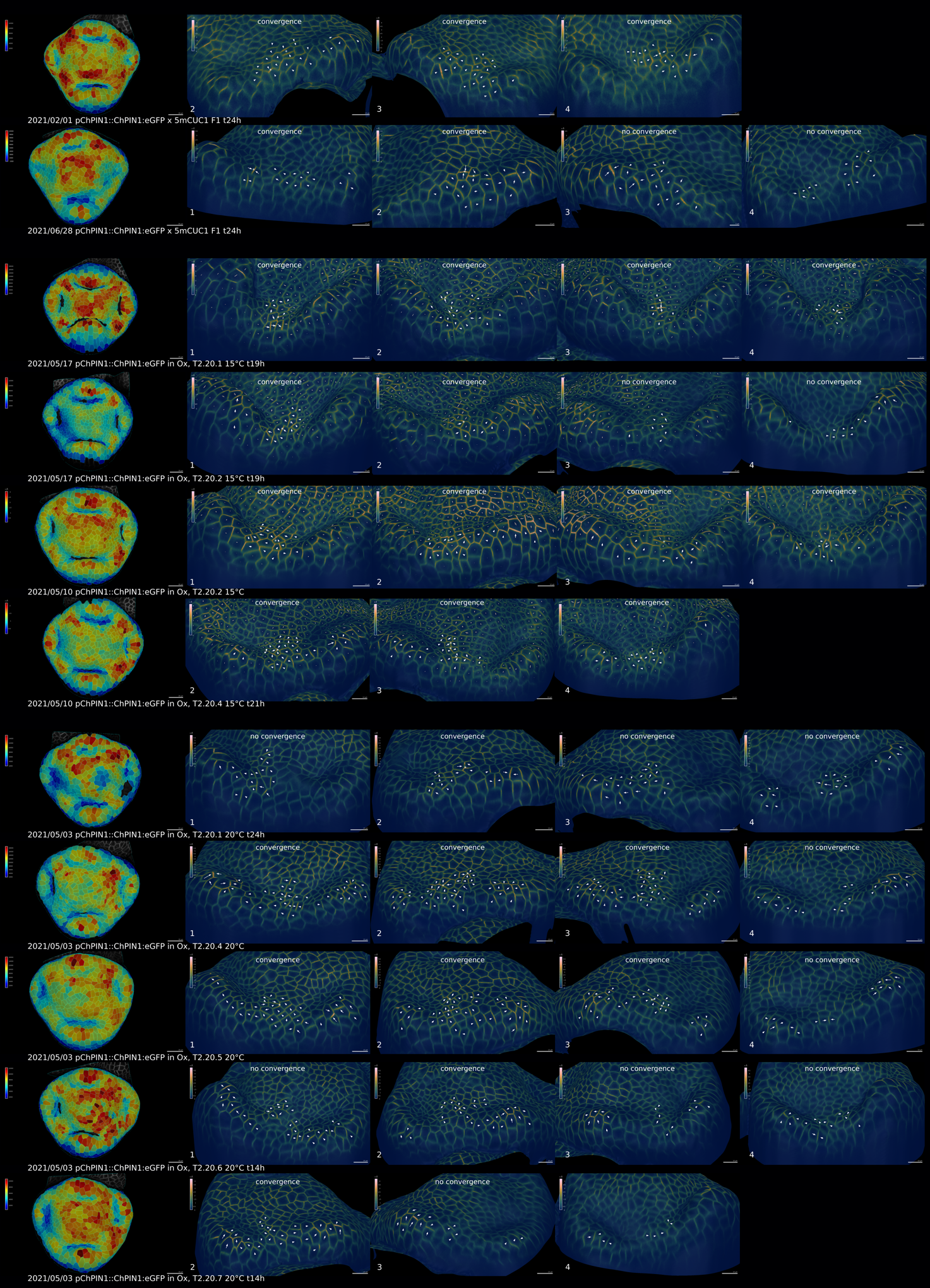
**

#### Fig. S2 *PIN1* expression pattern and plasma membrane localisation in stage 4 *C. hirsuta* flowers.

Additional replicates showing plasma membrane-localised signal of *pChPIN1::ChPIN1:eGFP* in inter-sepal regions of *C. hirsuta cuc2* (n = 1), *5mCUC1* (n = 2), WT grown at 15ºC (n = 4) and 20ºC (n = 5), shown in side view projections. Heat maps show epidermal signal intensity at the cell edges in arbitrary units. Arrows estimate cell polarity of the signal using vectors computed by MGX with arrowheads added manually to face the most intense signal. Our interpretation of whether PIN1 converges or not is written on each screenshot. Scale bars: shown on individual images. A high resolution version of this figure, as well as raw data, confocal stacks in tiff format, and MorphoGraphX segmentation meshes are available at <https://doi.org/10.17617/3.93>

####


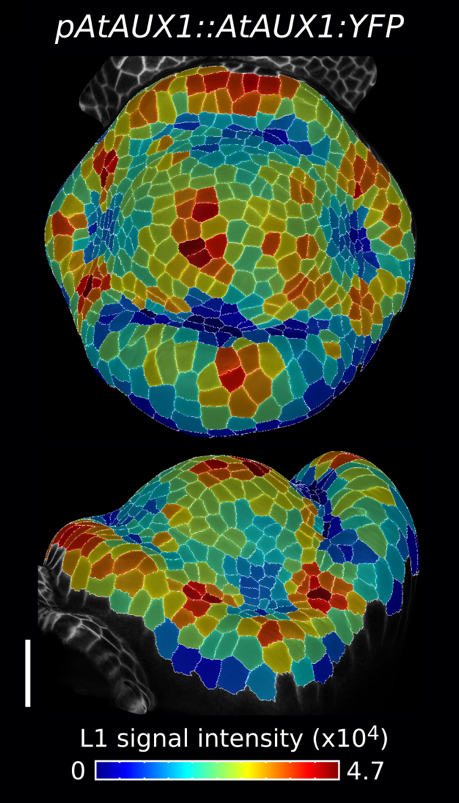


#### Fig. S3 AUX1 expression pattern in Arabidopsis. Expression of *pAtAUX1::AtAUX1:YFP* (n=7) in stage 4 floral primordia of Arabidopsis, shown in top and side view projections. Heat maps show average epidermal cell signal intensity in arbitrary units. Scale bar: 20 μm. No staining with PI, the segmentation is based on the AUX1 signal at the membrane.

**
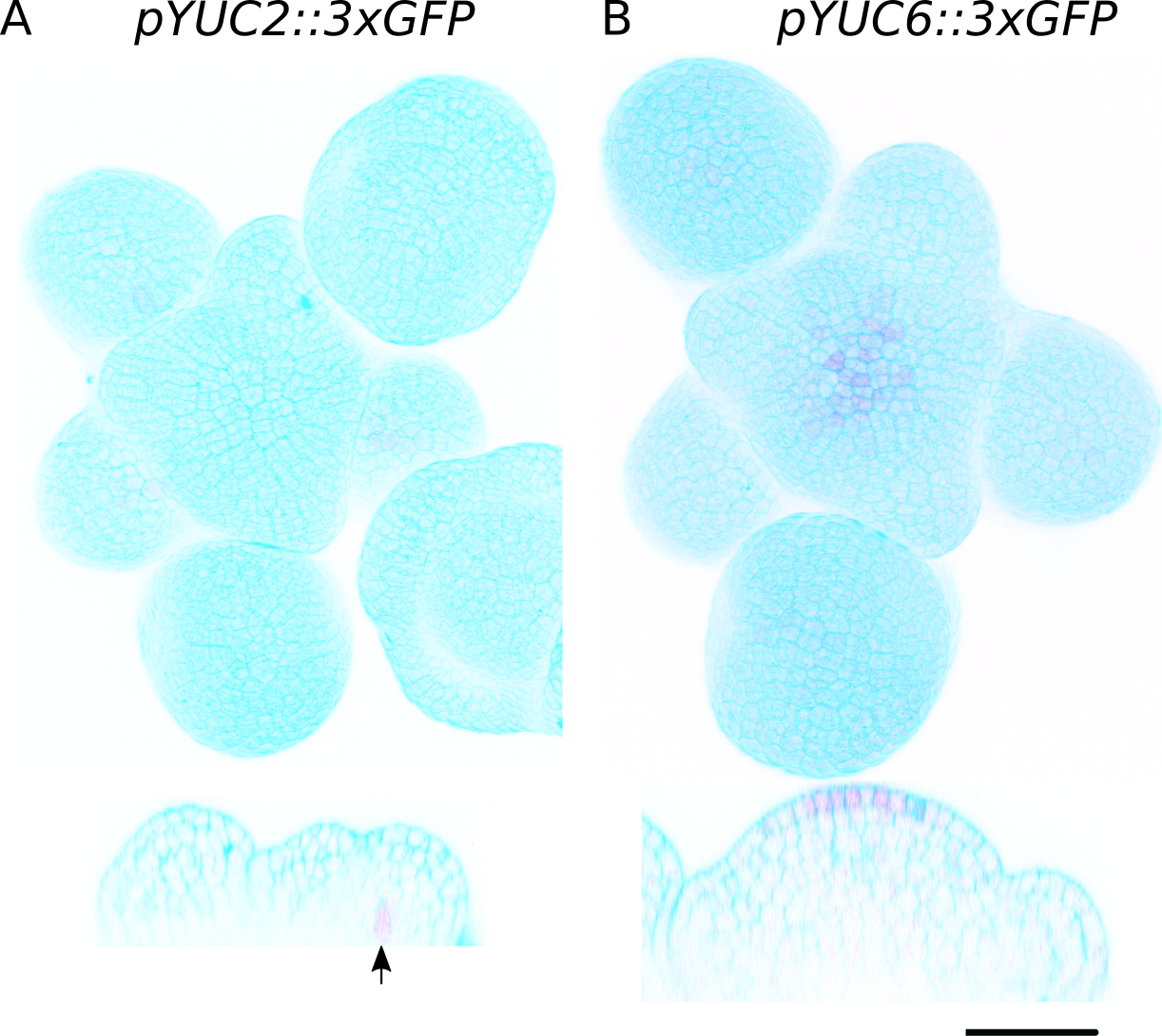
**

#### Fig. S4 Expression patterns of *YUC2* and *YUC6* in Arabidopsis inflorescences. Expression of *pYUC2::3xGFP* (A), and *pYUC6::3xGFP* (B) fluorescent reporters (magenta) and cell outlines (propidium iodide, cyan) in Arabidopsis. Top images are maximum intensity projections of the confocal stacks, and bottom images are orthogonal sections showing *YUC2* expression in internal tissues of a stage 3 flower bud (black arrow) and epidermal *YUC6* expression in the SAM. Scale bar: 50 µm.

####
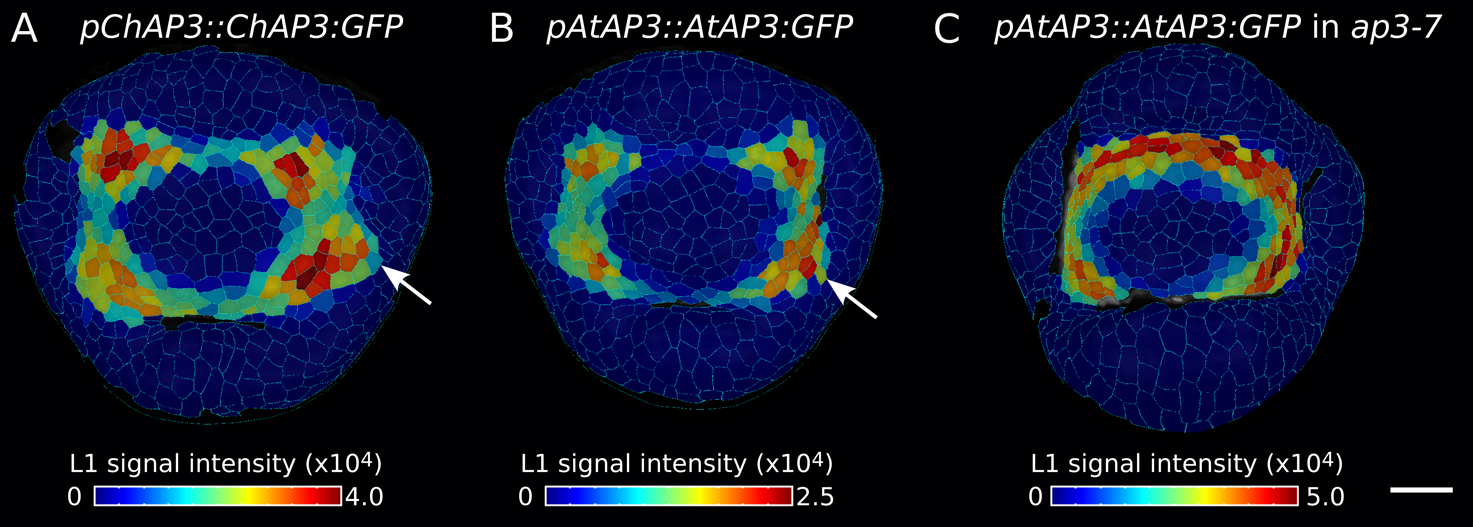


#### Fig. S5 *AP3* expression extends into inter-sepal regions in *C. hirsuta* flowers. Stage 4 flowers of *C. hirsuta* expressing *pChAP3::ChAP3:GFP* (A) and *pAtAP3::AtAP3:GFP* (B). Arrows point to a single inter-sepal region in each flower showing *AP3* expression extending into this region in *C. hirsuta* flowers. (C) Stage 4 flower of Arabidopsis *ap3-7* expressing the complementing *pAtAP3::AtAP3:GFP* construct. Heat maps show average epidermal cell signal intensity in arbitrary units. Scale bar: 20 μm.
